# Promoting anti-corruption, transparency and accountability in the recruitment and promotion of health workers to safeguard health outcomes

**DOI:** 10.1080/16549716.2019.1701326

**Published:** 2020-03-20

**Authors:** Monica Twesiime Kirya

**Affiliations:** U4 Anti-Corruption Resource Centre, Chr. Michelsen Institute, Bergen, Norway

**Keywords:** Anti-Corruption, Transparency and Accountability, Corruption, health workers, recruitment, promotion, health outcomes

## Abstract

**Background**: Human Resources for Health are a core building block of a health system, playing a crucial role in improving health outcomes. While the existing literature has examined various forms of corruption that affect the health sector, few articles have examined the role and impact of corruption in the recruitment and promotion of health-workers.

**Objectives**: This study reviews the role of corrupt practices such as nepotism, bribery and sextortion in health-worker recruitment and promotion and their implications for health systems.

**Methods**: The study is based on an interdisciplinary non-systematic review of peer-reviewed journal articles in the public health/medicine and political science literature, complemented with the ‘grey’ literature such as technical reports and working papers.

**Results**: Political and personal ties, rather than merit are often factors in the recruitment and promotion of health-workers in many countries. This results in the employment or promotion of poorly qualified or unsuitable workers, with negative implications for health outcomes.

**Conclusion**: Corrupt practices in health-worker recruitment and promotion ‘set the tone’ for other forms of corruption such as absenteeism, embezzlement, theft and bid-rigging to flourish, as those recruited corruptly can collude for nefarious purposes. On the other hand, merit-based recruitment is important for curbing corruption. Corrupt recruitment practices have deleterious effects on health-worker motivation and retention, quality and competency, citizens’ trust in health services and health outcomes. Whereas international law and policy such as the United Nations Convention Against Corruption and the WHO Handbook on Monitoring and Evaluation of Human Resources for Health state that recruitment of public officers and health workers respectively should be done in a transparent and accountable manner, more research is needed to inform policies on merit-based recruitment.

## Background

Anti-Corruption, Transparency and Accountability in all aspects of public service delivery, including health services, is a pre-requisite for human development and for building peaceful, inclusive societies with healthy populations. Substantially reducing corruption in all its forms and developing effective, accountable and transparent institutions at all levels are important targets in the Sustainable Development Goals [1] and are the subject of an international treaty, the United Nations Convention Against Corruption [2].

Corruption in Human Resources for Health (HRH) is a cause for concern in advancing the global health agenda because of its deleterious impact on access to and quality of healthcare, including its negative impact on morbidity and mortality [3,4]. Human Resources for Health in this paper refers to health service providers as well as people not engaged in the direct provision of services, referred to as ‘health management and support workers’ [5].

This paper focuses on corruption in recruitment and promotion because there is evidence, as will be discussed below, that it fosters other types of corruption in human resources for health. The traditional method for public service recruitment in many bureaucracies is to evaluate candidates based on formal examinations. Recruitment in the health sector follows a similar process, with health workers recruited based on passing the requisite examinations in medical schools and colleges [6].

There is substantial evidence that academic dishonesty and cheating in medical schools and colleges is common in many countries, with adverse implications for the quality of health workers [7–14]. This paper does not look at cheating by students in health colleges, but there is no doubt that it too, undermines the principle of merit-based recruitment of health workers.

Corruption in recruitment and promotion of health workers impacts the poorest and most vulnerable members of society, particularly in countries that are already perceived as highly corrupt, are undergoing skewed health worker migration (i.e. brain-drain), and that also suffer from poor population health outcomes [15]. Some elements of corruption in health worker management are absenteeism, ghost workers, conflicting dual public and private practice; as well as patronage, nepotism, cronysim as well as bribery and extortion to influence recruitment, posting, transfer and promotion decisions. Sextortion, in which sex is the currency of a bribe or extortionary transaction, is also recognised as abuse of power to obtain a private (sexual) benefit, and hence is a form of corruption [16].

This paper focuses on corruption in recruitment and promotion due to the risks to health outcomes posed by recruiting unqualified and incompetent workers [17], as well as the fact that this type of corruption has been found to foster other forms of corruption.

There are several global governance documents that reinforce the importance of tackling corruption in HRH. The aforementioned United Nations Convention Against Corruption, Article 7, enjoins States Parties to adopt, maintain and strengthen systems for the recruitment, hiring, retention, promotion and retirement of civil servants and other non-elected public officials that are based on principles of efficiency, transparency and objective criteria such as merit, equity and aptitude. The 2008 WHO Kampala Declaration states that Government leaders, Ministers of Health and other national leaders will commit to providing ‘all people, everywhere with access to a skilled, motivated and facilitated health worker within a robust health system.’ The Declaration further enjoins governments to ‘assure adequate incentives and an enabling and safe working environment for effective retention and equitable distribution of the health workforce’ [18]. Corruption in the recruitment and promotion of health workers poses a threat to these goals.

In addition to these instruments, the WHO Global Strategy on Human Resources for Health emphasises the importance of ensuring ethical recruitment practices [19]. The WHO Global Code of Practice on the international recruitment of health personnel states that recruitment should be transparent, fair and sustainable [20].

Separate from health governance and legal frameworks, the political science literature has considered how the politicisation of public service delivery jobs is a problem in many countries, with implications for efficiency and performance [21]. Politicisation is defined as ‘the substitution of political criteria for merit-based criteria in the selection, retention, promotion, rewards, and disciplining of members of the public service’ [22, p. 2]. In addition to politicisation, the literature also mentions the role of bribery, extortion and favouritism based on kinship (nepotism) or friendship (cronyism), in recruitment in the public sectors of various countries [23–25], and in the health sector in particular [26].

Researchers have found a co-relation between non-meritocratic recruitment and corruption in the public service [27]. In addition to recruitment, appropriate posting and transfer of healthcare workers is increasingly recognised as an important issue in HRH to promote health system strengthening (HSS) and citizens’ trust in government health services [28]. As mentioned above, corruption can also subvert posting and transfer decisions, negatively affecting health service delivery and population outcomes.

Despite the significant potentially negative consequences of corruption in health worker recruitment and promotion, there is limited research on the subject. This could be because it is not a big problem, because this form of corruption is more hidden and difficult to observe by respondents to health sector corruption surveys, or because surveys do not cover this aspect of health sector corruption.

One survey that did was the USAID Health Sector Corruption Survey 2013, which surveyed 1,125 health managers and leaders working in more than 95 countries. It reported that nepotism and favouritism in employment and promotion were the least-observed forms of corruption, whereas kickbacks and bribes were the most common [29]. However, the survey had several limitations, including a low response rate.

Accordingly, this article conducts an interdisciplinary review to shed further light on this problem. The review intersects between public health, political science, and more specifically HRH. Based on the findings, the study concludes that ethical and meritocratic recruitment and promotion in the health sector is indispensable for good health outcomes and needs further attention and research.

## Methods

A multi-disciplinary non-systematic literature review of the medical and political science literature was conducted. The search aimed to identify peer-reviewed on PubMed and JSTOR published between 2000–2018. Combinations of these search terms were used: ‘corruption + health sector jobs,’ ‘corruption + human resources for health,’ ‘corruption + health worker recruitment,’ ‘corruption + health worker promotion,’ ‘patronage + public sector jobs,’ ‘cronysim + public sector jobs,’ ‘favouritism + public sector jobs,’ ‘sexual harassment + health sector,’ and ‘merit + recruitment + public sector.’ The same keyword searches were used in Google scholar and the Google search engine to identify non-indexed articles, technical papers, policy documents, and other relevant materials. Duplicates were eliminated manually after compiling a working bibliography. Articles were selected for review based on their relevance after perusing abstracts. The final choice of papers reviewed were those that described or evaluated corrupt practices in public service recruitment and promotion generally, corruption in the recruitment and promotion of health workers specifically and those that mentioned merit-based recruitment.

## Results

### Typology of corrupt practices in health worker recruitment and promotion

A total of 49 peer reviewed articles were selected for review based on the criteria described above; these were supplemented by 18 working papers, book chapters and policy reports. This section describes the types of practices that occur and their impact of health workers and health outcomes (see Table 1 for summary).

**Table 1.** Types of corrupt practices in health worker recruitment and promotion.

Type of corruption	Definition
Patronage/Clientelism	Political support rewarded with public appointments and public sector jobs and promotions
Nepotism	Public officials exploit their positions of authority to favour relatives in obtaining jobs, advancement, or other preferential treatment.
Cronyism	Public officials exploit positions of authority to favour their friends and associates in obtaining jobs or advancement
Bribery	Payment of fees or giving gifts in exchange for passing recruitment examinations, being shortlisted, being interviewed, being appointed to the job or being promoted.
Extortion	Bribery accompanied by a threat from the bribe-taker
Sextortion	Bribery where sex is the currency of exchange

### Patronage and clientelism

Patronage is often conceptualised alongside clientelism and both refer to the exchange of political support for favours such as jobs, contracts or public goods [30]. It involves a relationship between a politician and a supporter or group of potential supporters, in which the politician uses their public position to compensate and reward individuals or groups who have played an important role in their election or ascension to power; often the reward is granted in the form of access to a salaried public- sector job. In this manner, members of certain political groups are given preference when it comes to filling positions in the public sector [31].

Patronage and clientelism are common in many countries; indeed, Mungiu-Pippidi argues that such particularistic governance practices are the default rather than the exception in countries where resources are scarce [32]. Similarly, Khan avers that patronage and clientelism are prevalent in developing countries due to the nature of the political settlement. The term ‘political settlement’ is used to describe the ‘social order’ based on political compromises between powerful groups in society that sets the context for institutional and other policies. Informal means of distributing benefits such as jobs prevail due to resource scarcity [33].

Patronage is regarded by some scholars as politically legitimate in the appointment of senior personnel such as Ministers and senior bureaucrats. It is thought that political leaders rely on it to ensure that key decision-making positions are staffed by administrative officials whose policy preferences and priorities are like their own to ensure that their policies will be implemented [34].

However, when political affiliation rather than merit is a common factor in recruitment at all levels of the bureaucracy, it is problematic because of its inherently discriminatory nature. It is even worse when patronage is involved in the recruitment or promotion of frontline health workers who handle patients because it does not prioritise competency and thus bears direct implications for health outcomes.

The problem of patronage in health sector recruitment has been examined in several studies in the medical field. Evaluations from Afghanistan and Rwanda reported that patronage and nepotism hindered efforts to reform and restructure their health sectors during their respective post-conflict reconstruction efforts [35,36]. Abimbola et al.’s study in Nigeria reported that HRH management decisions in the health service were sometimes based on requests for favours from health workers and politicians. In addition, lobbying powerful political intermediaries could be used by health workers to reverse a decision they deemed unfavourable [37]. Karakose’s study in Turkey interviewed doctors on their views on favouritism and reported that unfairness in appointing managers based on their political and ideological views was common [38]. La Forgia et al.’s qualitative study on the Dominican Republic stated that patronage was a key factor in the appointment and deployment of health staff, with recommendations from politicians, political parties, military officials and top governmental officials influencing decisions [39]. A research brief by Camargo on Uganda revealed that there are widespread informal power networks that influence decision-making in the health sector, especially at decentralised government level. The extensive patronage network enabled health workers who had powerful godfathers to be recruited, and once recruited, they could get away with indiscipline because of their political connections [40]. Patronage and clientelism can thus have a deleterious impact on professionalism in the health workforce, quality of health services, and equity in the geographical distribution of health workers.

### Nepotism and cronyism

Nepotism is a term used to describe situations in which elected or appointed public officials exploit their positions of authority to favour relatives in obtaining jobs, advancement, or other preferential treatment [41]. This is often done in a manner that overlooks professional job requirements. Cronyism means, ‘the appointment of friends and associates to positions of authority, without proper regard to their qualifications,’ [42].

Studies that have considered nepotism and cronyism in the health sector include Abimbola’s study on Nigeria referred to above, which reported that Senior Medical Personnel personalized appointments outside formal procedures and without clear criteria, ‘because of gratitude or friendship’ [37]. This sometimes resulted in the recruitment of persons who displayed signs of incompetency. Karakose’s enquiry into Turkish doctors’ views on favouritism reported that prioritising friendship relationships in decision-making was a common practice potentially resulting in perceptions of unfairness and concomitant dissatisfaction of health workers [38]. Similarly, in Uganda, Carmago reported that personal connections were an important factor in the recruitment and promotion of health staff and that promotion was dependent on continued demonstration of loyalty to the appointing patron by participating in other corrupt acts such as demanding informal payments (bribery) and embezzlement [43]. These examples show how cronyism and nepotism can undermine the equitable recruitment and promotion of competent workers, with profound effects for health care as will be discussed below.

### Bribery and extortion

Bribery and extortion in recruitment is also referred to as job purchasing [44]. Blunt et al.’s study on Indonesia reported that bribery and extortion sometimes occurred in health sector recruitment [45]. It involved offers and demands for payment of fees to pass public service recruitment exams, payment of fees to be shortlisted, to be interviewed, to secure a job, to secure a posting considered favourable due to locality or organisation and even to be confirmed after a probationary period or to be promoted [45]. According to Harris et al, paying for posts was also common in Nepal [46]. Naher et al.’s aforementioned study on health sector corruption in Bangladesh showed that corruption is incentivised at the beginning of a medical career, when students have to pay large ‘donations’ to be admitted to private medical colleges. In addition, directors of district hospitals abuse their autonomy in the recruitment and promotion of healthcare staff by accepting bribes for the transfer and promotion of staff. The situation is exacerbated by weak or non-existent regulations, lack of accountability, low salaries and limited opportunities for promotion [14].

### Sextortion

Whereas sexual harassment has long been recognised as a problem in human resources, it is only recently that it has been regarded as a type of corruption, depending on certain conditions elaborated below. The International Association of Women Judges (IAWJ) pioneered this view in 2012, stating that ‘sextortion is a form of corruption in which sex, rather than money, is the currency of the bribe.’

The IAWJ emphasises that-

There are many forms of sexual abuse, harassment, exploitation, and discrimination – all of which are of serious concern to people who care about human rights and gender equality, and all of which need to be addressed and ended. What distinguishes sextortion from other types of sexually abusive conduct is that it has both a sexual component and a corruption component. Conduct that does not include both components *is not* sextortion [16, p. 9].

This study’s literature search turned up substantial evidence of sexual harassment of health workers especially female nurses, although the link to corruption in employment or promotion was implicit and not explicit [47–54]. A 2014 quantitative review of studies on sexual harassment of nurses turned up a total of 136 articles with data on 151,347 nurses from 160 samples and revealed that up to 25% of nurses have experienced sexual harassment at work [55]. This shows the potential scale of the problem. One study mentioned that health workers were required to pay or ‘offer personal services’ to have their papers or pay cheques processed [56]. Managers and supervisors have been implicated in sexual harassment of nurses and other health workers, indicating an abuse of power [57]. However, more research and analysis linking sexual harassment of health workers to corruption and the abuse of power is needed.

The various forms of corruption above have negative effects on the health sector. Corrupt practices in recruitment and promotion ‘set the tone’ for other forms of corruption such as absenteeism, embezzlement, theft and bid-rigging to flourish, as they may enable colleagues recruited corruptly to collude and connive for nefarious purposes. They also breed impunity because errant health workers with connections to powerful people are difficult to discipline. In addition, corrupt recruitment practices have deleterious effects on health worker motivation and retention, undermine citizens’ trust in health services and have adverse effects on health outcomes. These are elucidated further below.

## The impact of corruption in health worker recruitment and promotion

### Impact on health sector transparency and accountability

Corruption in the recruitment and promotion of health workers has several implications for the health sector. Few studies specifically examine the impact of specific forms of corruption such as patronage, nepotism and favouritism on health service delivery and health outcomes; most existing studies look at the impact of corruption generally. Nonetheless, Baez-Carmago’s research report on Uganda shows how patronage and clientelism in the appointment of public servants increase the likelihood of establishing and perpetuating networks of individuals within organisations who may connive and collude to embezzle public funds, engage in absenteeism or divert supplies and equipment for their own benefit, whilst covering up for each other [43]. Naher et al. posit that when medical students must pay large bribes for admission to college, sometimes necessitating families selling or mortgaging their land or obtaining credit, when they graduate and become poorly paid health professionals, they have little choice but to earn extra money through informal (corrupt) means by accepting informal payments (bribes) [14].

Baez-Carmago and Sambaiga’s research in Tanzania illustrates how the exchange of political support for public positions embeds a culture of reciprocity that rationalises bribery in the form of informal payments and extortion of citizens who must then access public services not as a right, but on a transactional basis [58]. Baez-Carmago argues that within the health sector, corruption by health ministers and government officials is imitated by administrators of public hospitals, doctors, nurses and other healthcare personnel, entrenching the vicious cycle of corruption across sectors including health [43]. A similar observation was made by Mostert et al.’s study on corruption and its effect on cancer care in Africa. ‘Corrupt misconduct of health ministers and government officials is imitated by the administrators of public hospitals. Likewise, doctors are corrupt, which allows nurses and other health-care personnel to misuse their power for private gain, allowing a culture of corruption to become widespread and accepted’ [59, p. e400].

### Impact on professional competency standards

Corruption in the recruitment or promotion of health workers undermines professional competency standards and disciplinary systems by encouraging unclear and opaque criteria based on informality, thus undermining formal standards and procedures [60]. In such situations, sanctioning errant health workers who have personal and kinship ties to powerful people becomes difficult or even impossible [43,61]. Moreover, informal ties between health workers, politicians and supervisors may lead to disciplinary actions being undertaken in a non-transparent manner either to obscure wrong-doing or as vengeance against opponents and those who resist corruption [62].

### Impact on motivation of health workers

Low motivation has been identified by some studies as the second most important health workforce problem after staff shortages [63]. Corruption in the recruitment and promotion of health workers may affect their motivation and retention [64,65]. However, while there are several studies on health worker motivation, few explicitly mention corruption in HRH as a factor. One study mentions performance management as a factor in health worker motivation and notes that corruption makes it difficult to implement performance management by undermining objective criteria for the assessment of performance, thereby affecting health worker motivation [63]. Political interference has also been identified as a factor that affects health worker morale and results in the attrition of health workers from public to private sector health facilities and causes them to migrate to more developed countries [66].

Corrupt practices involving sextortion have a pernicious effect on the work environment and job satisfaction [67]. Since there were no studies identified using the term ‘sextortion’ in the health workforce, the results presented here are extrapolated from studies on sexual harassment of health workers. The studies show that sexual harassment of nurses negatively affects the work environment and functioning in the workplace [67–72].

### Impact on health outcomes

The literature search did not turn up many studies that specifically analysed the impact of corruption in recruitment, and promotion on health outcomes. Nonetheless, it can be reasonable inferred from the literature that corruption in decisions regarding posting and transfer of health workers can undercut the equitable and effective delivery of health care, as health workers can use corrupt means to ensure that they are recruited to work in the urban areas that they consider more lucrative [17]. ‘Lucrative’ often refers to health workers’ ability to extract informal payments or bribes from patients to supplement their income [46] and to refund themselves for the money they used to bribe and influence their recruitment and posting [14] . Research shows that the effect of informal payments on the quality and accessibility of health care is complex [73], but they can deter access to health care, reduce the demand for health care, and affect the quality of care offered, especially for low-income groups [73,74].

Corruption poses a direct threat to patients’ health when those who are recruited are ill-qualified and not competent to do their jobs properly. Most of the identified literature on the link between corruption and health outcomes is based on a general comparison of data sets, for example, comparing child mortality rates with the corruption perceptions index (CPI) [75], or world health survey data with the CPI [76], or antibiotic consumption with the CPI [77]. From these studies and based on Mungiu-Pippidi [33] and Khan’s [34] ideas about the prevalence of particularism and patronage in developing countries, we can infer a significant likelihood that some health workers are hired in non-transparent ways that disregard merit. Health workers hired corruptly are more likely to be inexperienced, incompetent and poorly motivated, thus affecting the quality of health care and concomitant health outcomes. Indeed, Mostert et al.’s study on the effect of corruption on cancer care posits that corruptly recruited, inexperienced medical staff provide sub-standard cancer-care due to difficulties in the application of complex chemotherapeutic protocols and that displays of incompetence and corrupt behaviour cause patients and communities to mistrust and disrespect health personnel, which may reduce adherence to treatment, stimulate the abandonment of treatment, increasing rates of morbidity and mortality [59].

## Discussion

Corruption in the recruitment and promotion of health workers is common in many countries and has negative implications for health service delivery and health outcomes. While there are other forms of corruption in human resources for health such as absenteeism, conflicts of interest and theft, some of the studies above [43,59] show that corruption in recruitment and promotion foments and exacerbates these other forms of corruption. Worryingly, it results in the recruitment of unqualified and incompetent personnel who may not be able to implement complex treatment protocols properly [59].

On the other hand, a study by Dahlstrom et al. [77] using a regression analysis reported that meritocratic recruitment reduces corruption, whereas other relevant bureaucratic factors such as public employees’ competitive salaries, career stability, or internal promotion do not have a significant impact on corruption. The evidence of the interrelationship between merit-based recruitment and corruption underscores the importance of addressing this issue. It is important that countries adopt merit-based recruitment systems based on objective criteria not just to ensure that properly qualified and skilled HRH, but also to curb other types of corruption in the sector.

The global community recognises the importance of this and enshrines the principles of transparency and fairness in recruitment as a key aspect of the WHO Global Code of Practice on the international recruitment of health personnel [20]. The Code enjoins WHO Member States to take effective measures to educate, retain and sustain a health workforce that is appropriate for the specific conditions of each country, including areas of greatest need, and is built upon an evidence-based health workforce plan. This in turn requires the establishment and maintenance of efficient health personnel information systems, with accurate information on the health labour market. However, there are no specific detailed guidelines for transparent and accountable recruitment in the sector. Indeed, the literature search did not turn up much in the way of specific guidelines on merit-based recruitment for the public sector in general or for HRH in particular.

## Conclusion

Measures to promote anti-corruption, transparency and accountability in recruitment and posting decisions are necessary to safeguard population health outcomes. Merit-based recruitment of health workers is an important foundation of health systems, a guarantor of improved health outcomes and a proven way of curbing corruption in the public service. The studies reviewed here show that it is a significant problem in some countries. More empirical research at country level is needed to enable evidence-based context-specific solutions. Health system assessments undertaken as part of health sector planning should establish the extent to which corruption is a problem is the recruitment and deployment of health workers and whether there are policies to address the problem, as recommended by the WHO [78]. In addition, international organisations such as the WHO and national governments should ensure that more detailed guidance on recruitment and promotion is published and that it is followed.

## Disclaimer

This article represents the views of the author and in no way should be interpreted to represent the views of, or endorsement by, her affiliated institutions(s), U4 Anti-Corruption Resource Centre Partner Agencies, or of the World Health Organisation as a sponsor of this GHA Special Edition on ‘Anti-Corruption, Accountability and Transparency in the Health Sector.’ The World Health Organisation shall in no way be responsible for the accuracy, veracity and completeness of the information provided through this article.

## Paper context

Despite recognition that Human Resources for Health are a crucial factor in health systems strengthening, there are few studies on the extent to which corrupt practices influence recruitment and promotion decisions in the health sector and the implications for health outcomes. This review summarises the findings from existing studies and shows that more research is needed to inform health systems assessments, plans and policies and formulate evidence-based policies for merit-based recruitment and promotion of workers.
